# Evasion of IFN-γ Signaling by *Francisella novicida* Is Dependent upon *Francisella* Outer Membrane Protein C

**DOI:** 10.1371/journal.pone.0018201

**Published:** 2011-03-31

**Authors:** Kalyan C. Nallaparaju, Jieh-Juen Yu, Stephen A. Rodriguez, Xhavit Zogaj, Srikanth Manam, M. Neal Guentzel, Janakiram Seshu, Ashlesh K. Murthy, James P. Chambers, Karl E. Klose, Bernard P. Arulanandam

**Affiliations:** South Texas Center for Emerging Infectious Diseases and Department of Biology, University of Texas at San Antonio, San Antonio, Texas, United States of America; Louisiana State University, United States of America

## Abstract

**Background:**

*Francisella tularensis* is a Gram-negative facultative intracellular bacterium and the causative agent of the lethal disease tularemia. An outer membrane protein (FTT0918) of *F. tularensis* subsp. *tularensis* has been identified as a virulence factor. We generated a *F. novicida* (*F. tularensis* subsp. *novicida*) FTN_0444 (homolog of FTT0918) *fopC* mutant to study the virulence-associated mechanism(s) of FTT0918.

**Methods and Findings:**

The *ΔfopC* strain phenotype was characterized using immunological and biochemical assays. Attenuated virulence via the pulmonary route in wildtype C57BL/6 and BALB/c mice, as well as in knockout (KO) mice, including MHC I, MHC II, and µmT (B cell deficient), but not in IFN-γ or IFN-γR KO mice was observed. Primary bone marrow derived macrophages (BMDM) prepared from C57BL/6 mice treated with rIFN-γ exhibited greater inhibition of intracellular *ΔfopC* than wildtype U112 strain replication; whereas, IFN-γR KO macrophages showed no IFN-γ-dependent inhibition of *ΔfopC* replication. Moreover, phosphorylation of STAT1 was downregulated by the wildtype strain, but not the *fopC* mutant, in rIFN-γ treated macrophages. Addition of N^G^-monomethyl-L-arginine, an NOS inhibitor, led to an increase of *ΔfopC* replication to that seen in the BMDM unstimulated with rIFN-γ. Enzymatic screening of *ΔfopC* revealed aberrant acid phosphatase activity and localization. Furthermore, a greater abundance of different proteins in the culture supernatants of *ΔfopC* than that in the wildtype U112 strain was observed.

**Conclusions:**

*F. novicida* FopC protein facilitates evasion of IFN-γ-mediated immune defense(s) by down-regulation of STAT1 phosphorylation and nitric oxide production, thereby promoting virulence. Additionally, the FopC protein also may play a role in maintaining outer membrane stability (integrity) facilitating the activity and localization of acid phosphatases and other *F. novicida* cell components.

## Introduction

*Francisella tularensis*, a Gram-negative facultative intracellular bacterium, causes the disease tularemia [1]. Of the *F. tularensis* subspecies (subsp) described to date, those responsible for human disease include the highly virulent subsp. *tularensis* (type A) and the moderately virulent subsp. *holarctica* (type B) [2]. Although *Francisella novicida* (also referred to *F. tularensis* subsp. *novicida* [3]) is avirulent in humans, it behaves similarly to *F. tularensis* in human and mouse macrophages [4], shares biochemical features with *F. tularensis* [3], [5] and exhibits an LD_50_ in mice similar to *F. tularensis* via the pulmonary route [2], [6]. There also are notable differences between *F. novicida* and *F. tularensis*, such as the LPS of *F. novicida* being more stimulatory [7], [8], [9]. However, *F. novicida* has been an attractive model for the study of the virulence mechanisms of *F. tularensis*, given the genetic similarity (98.1% homology between sequences common to U112 and SCHU S4) to *F. tularensis* [10], and similar lifestyle within host cells.

*Francisella* can infect a variety of cell types including pulmonary epithelial cells [11], [12], dendritic cells [13], and hepatocytes [14], but exhibits a strong tropism for replication within macrophages [13]. The bacterial components responsible for the marked virulence of *Francisella* are still largely unknown. Among the most studied virulence factors are the *Francisella* pathogencity island (FPI) genes, which encode a secretion system required for phagosome escape and intramacrophage growth [15]. Additional virulence factors have been identified, in particular a 58 kDa outer membrane lipoprotein (FTT0918). This protein is truncated (expressed as a fusion protein) in the live vaccine strain (LVS) derived from *F. tularensis* subsp. *holarctica* [16], and reintroduction of FTT0918 into LVS restored virulence to a level indistinguishable from a wildtype subsp. *holarctica* strain [17]. Moreover, deletion of FTT0918 in *F. tularensis* subsp. *tularensis* also attenuates virulence [16]. Given the importance of this outer membrane lipoprotein (designated FopC) in *F. tularensis* virulence and pathogenicity, we generated a defined *F. novicida* FTN_0444 (homolog to FTT0918, 99% identity in aa sequence) mutant, designated KKF332, and characterized the contribution of this protein to the virulence of this microorganism. Our results demonstrate that FopC plays a role in inhibition of IFN-γ-mediated host immune defense(s), thereby promoting virulence.

## Results

### Construction of the *F. novicida fopC* mutant, KKF332

**Figure 1:**
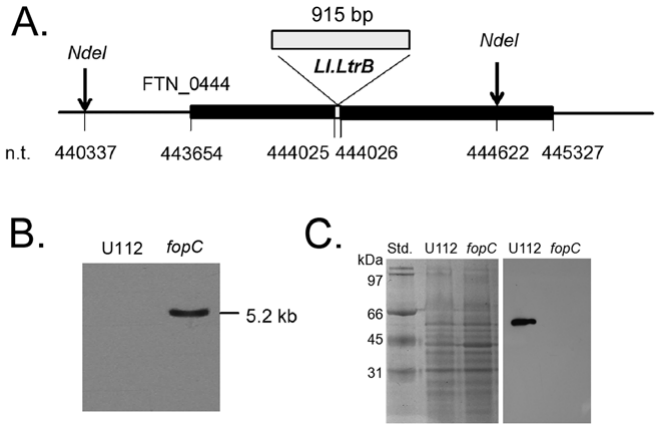
Generation of the *fopC*-null mutant (KKF332) of *F. novicida* U112. (A) Schematic representation of Ll.LtrB intron insertion in the *fopC* locus (FTN_0444) of KKF332. (B) Total genomic DNA, from wildtype U112 and insertion *fopC* mutant strain (FTN_0444:: Ll.LtrB) cells, was digested with NdeI and blots were probed using an intron-specific probe. The number to the right of the panel indicates the molecular mass in kilobases based on DNA standards. (C) U112 and *fopC* strain cell lysates were separated by 10% SDS-PAGE, transferred onto a PVDF membrane, and probed with polyclonal FopC antisera.

In order to evaluate the role of FopC protein (FTN_0444) as a virulence factor, we generated a *fopC* mutant of *F. novicida* using a previously described Targetron insertion method [18]. Site-specific insertion of a 0.9 kb-Ll.LtrB intron between nt. 444025 and 444026 of the *F. novicida* U112 genome resulted in a *fopC* mutant, designated here as KKF332. Schematic representation of the mutated *fopC* locus in KKF332 is shown in Fig. 1A. Disruption and inactivation of the *fopC* gene in KKF332 was confirmed by Southern and Western blot analyses, respectively. Southern blot analysis (Fig. 1B) using an Ll.LtrB intron-specific probe [18] detected a 5.2 Kb band which corresponded to the NdeI restriction digestion product of the KKF332 *fopC* gene locus, but not to the parental U112 strain. Western blot analysis (Fig. 1C) using murine anti-FopC polyclonal antibodies demonstrated the presence of a 58 kDa band in wildtype U112 cells, but not in *fopC* mutant cells further confirming the lack of FopC protein expression in KKF332.

### Virulence of the *fopC* mutant is attenuated in a murine pulmonary tularemia model

**Figure 2:**
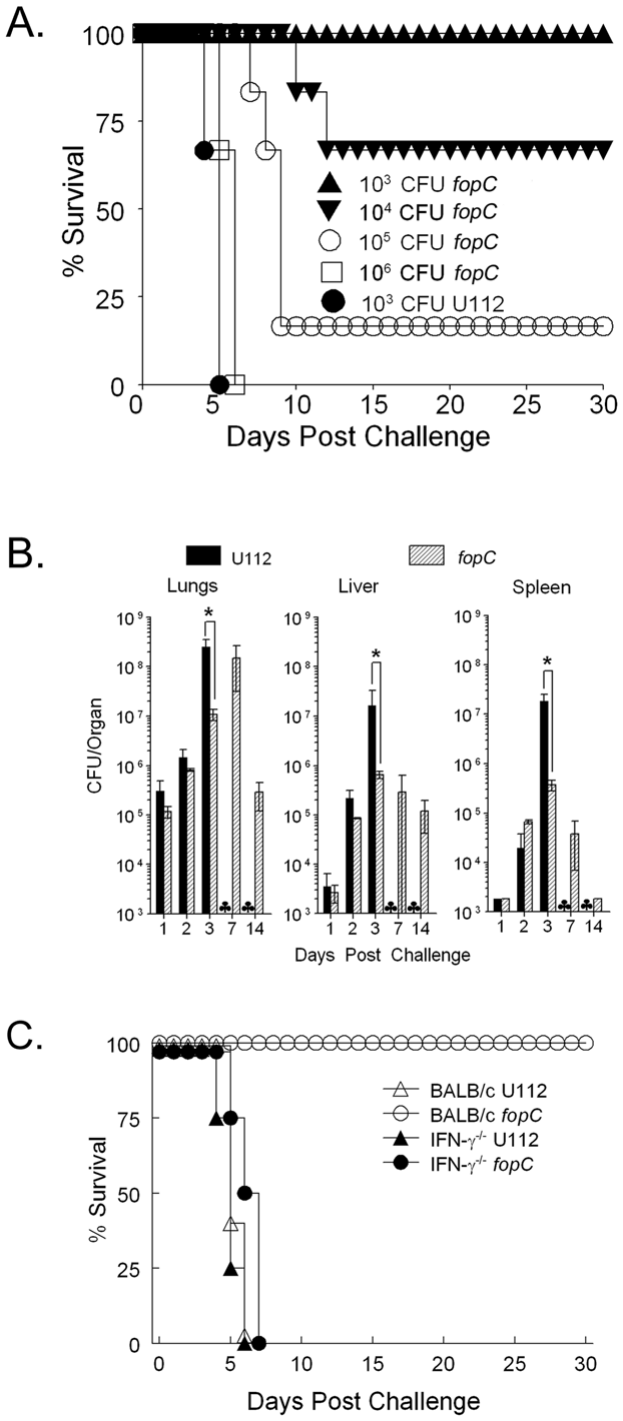
Virulence of the *fopC* mutant. (A) BALB/c mice (6/group) were challenged i.n. with 10^3^ CFU U112 or increasing inocula (10^3^–10^6^ CFU) of *fopC* mutant. All animals were monitored daily for survival for 30 days. (B) BALB/C mice were challenged i.n. with 10^3^ CFU wildtype U112 or *fopC* mutant. Organs (lungs, liver and spleen) were collected on days 1, 2, and 3 from mice (n = 3 mice at each time point) infected with U112 and on days 1, 2, 3, 7 and 14 postchallenge from mice infected with the mutant, homogenized, and dilution plated for bacterial enumeration. Results are presented as log_10_ CFU per organ (lower detection limit was approximately 1000 CFU/organ). Differences between replication of U112 and *fopC* strain on day 3 are significant with P values <0.05 represented by * in the figure. Death by day 7 of all mice resulting from U112 challenge is represented by ♣. (C) BALB/c IFN-γ^+/+^ and IFN-γ^−/−^ mice (8/group) were challenged i.n. with 10^3^ CFU wildtype U112 or *fopC* mutant. All animals were monitored daily for survival over 30 days. Difference in survival between *fopC* mutant challenged BALB/c and IFN-γ^−/−^ mice was significant at a *p*<0.001. Difference in survival between *fopC* mutant and U112 wildtype challenged BALB/c mice was significant at a *p*<0.001. (Representative of two experiments.)

The *fopC* mutant was assessed for virulence in a murine pneumonic tularemia model. To determine the LD_50_ of the *fopC* mutant using this model, BALB/c mice (6/group) were inoculated intranasally with an increasing dosage of strain KKF332. As shown in Fig. 2A, the LD_50_ of the *fopC* mutant was between 10^4^ and 10^5^ CFU, which was approximately 1000 fold greater than that of the wildtype U112 strain (∼10 CFU or less; [19]). As expected, all mice inoculated intranasally with 10^3^ CFU of U112 (∼100 LD_50_) succumbed to infection by day 5.

To further examine replication and dissemination of the *fopC* mutant in comparison to the wildtype *F. novicida* strain, BALB/c mice were challenged i.n. with 10^3^ CFU of strains KKF332 or U112, and bacterial burdens in the lungs, liver and spleen determined. Both *fopC* mutant and wildtype strain recovery were comparable in the lungs of infected mice at 1–2 days after inoculation and both strains also could be recovered at similar levels in the liver and spleen on the second day after challenge (Fig. 2B). However, on day 3, bacterial burdens were significantly (*p*<0.05) lower (1–2 logs) in all organs (lungs, liver and spleen) of *ΔfopC* infected mice in contrast to mice infected with the wildtype strain, which all succumbed to infection by day 5. In contrast, all *fopC* mutant infected mice survived and the organism was recovered at in increasing number in the lungs until day 7, with the number of recovered bacteria decreasing at day 14. Additionally, decreasing *fopC* mutant burdens were observed in the liver and spleen between day 3 and day 14. Collectively, reduced mortality and decreased bacterial burdens in the lungs, liver, and spleen demonstrate that *fopC* mutant virulence is attenuated, and that host immune responses could largely control mutant strain replication.

### IFN-γ mediates *fopC* mutant attenuation in mice

**Figure 3:**
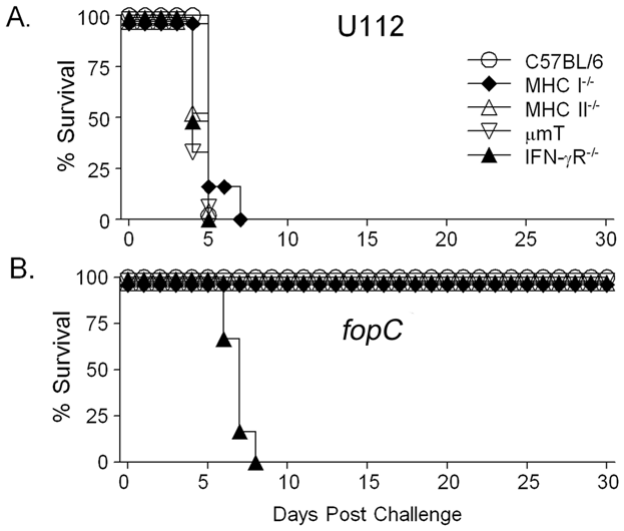
Susceptibility of knockout mice to wildtype and *fopC* mutant challenge. Different KO strains of mice (6/group) were challenged i.n. with 10^3^ CFU wildtype U112 (A) or *fopC* mutant (B). Difference in survival between *fopC* mutant challenged C57BL/6 and IFN-γR^−/−^ mice was significant at a *p*<0.001. All animals were monitored daily for survival over 30 days. (Representative of two experiments.)

Since IFN-γ has been shown to be a predominant component of the anti-*Francisella* protective immune response [20], [21], we compared survival of BALB/c IFN-γ^+/+^ and IFN-γ^−/−^ mice following i.n. challenge with 10^3^ CFU wildtype U112 or *fopC* mutant. As shown in Fig. 2C, both IFN-γ^+/+^ and IFN-γ^−/−^ mice challenged with U112 succumbed to infection within 6 days following challenge. However, only IFN-γ^−/−^, not IFN-γ^+/+^, mice succumbed within 7 days following *ΔfopC* challenge. BALB/c IFN-γ^+/+^ mice challenged with *ΔfopC* survived for at least 30 days following challenge. These results suggest that FopC may be required to mediate resistance of *F. novicida* to elimination by endogenous IFN-γ. We also analyzed whether mice deficient in endogenous IFN-γ signaling (IFN-γR^−/−^ mice) were susceptible to KKF32 challenge similar to the IFN-γ^−/−^ mice. Moreover, we used mice deficient in β2-microglobulin (MHC I^−/−^ mice), MHC II (MHC II^−/−^ mice), or B cells (µmT mice), all lacking components that have been shown to contribute to inducible IFN-γ production. We challenged C57BL/6 and different knockout (KO) mouse strains (MHC-I^−/−^, MHC-II^−/−^, µmT and IFN-γR^−/−^) intranasally with 10^3^ CFU 112 (Fig. 3A) or *fopC* mutant (Fig. 3B). All strains of mice succumbed to wildtype U112 infection by day 6. In contrast, the C57BL/6 mice and three of four KO mouse strains (MHC-I^−/−^, MHC-II^−/−^, and µmT) infected with the *fopC* mutant all survived for the entire period of the experiment (30 days). The notable exception was IFN-γR^−/−^ mice, which all succumbed to infection with the *fopC* mutant by 8 days.

**Figure 4:**
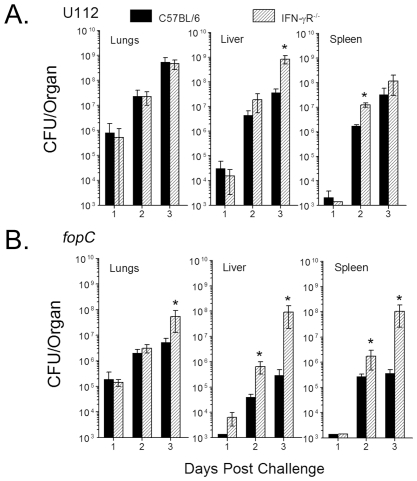
Dissemination of U112 and *fopC* mutant in C57BL/6 and IFN-γR^−/−^ mice. Mice were challenged i.n. with 10^3^ CFU wildtype U112 (A) or *fopC* mutant (B). Organs (lungs, liver and spleen) were collected on days 1, 2, and 3 postchallenge (n = 3 at each time point), homogenized, and dilution plated for bacterial enumeration. Results are log_10_ CFU per organ (lower detection limit was approximately 1000 CFU/organ). Differences between replication of U112 and *fopC* strains are significant with *p* values <0.05 represented by * in the figure. (Representative of two experiments.)

Examination of replication and dissemination profiles indicated wildtype U112 strain replication and kinetics of dissemination to the liver and spleen were similar in C57BL/6 and IFN-γR^−/−^ mice; although, higher (1–1.5 log_10_) numbers of bacteria were recovered from the spleen and liver of IFN-γR^−/−^ mice at days 2 and 3 (Fig. 4A). In comparison, the *fopC* mutant could be detected in the liver of IFN-γR^−/−^ mice one day earlier than in the liver of C57BL/6 mice, and bacterial loads were significantly higher (1–2.5 log_10_) in all tissues of IFN-γR^−/−^ mice at day 3 post challenge. Collectively, increased susceptibility of IFN-γR^−/−^ mice to the *fopC* mutant suggests that IFN-γ was one of the host components responsible for the attenuation of KKF332. These results demonstrate that IFN-γ signaling may play an important role in controlling the clearance of the *fopC* mutant following bacterial infection. Moreover, these findings are also in agreement with our previous observations [22] showing that CD4^+^ T cells are not required in the effector phase of antibody-mediated IFN-γ-dependent anti-*Francisella* immunity. Thus, the primary source of IFN-γ to confer protective immunity against the *fopC* mutant may come from innate immune cells such as macrophages and natural killer cells.

### IFN-γ mediates inhibition of replication of the *fopC* mutant within primary macrophages

**Figure 5:**
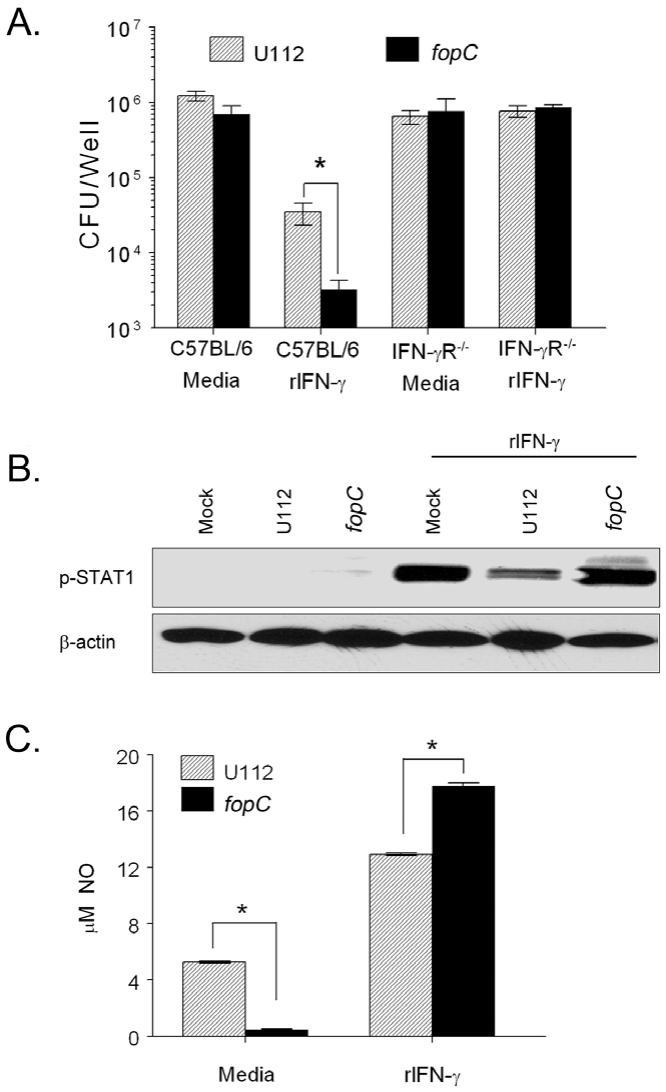
IFN-γ mediated inhibition of intramacrophage *F. novicida* replication. (A) Bone marrow derived primary macrophages (2×10^5^/well) from C57BL/6 or IFN-γR^−/−^ mice were infected (10 MOI) with wildtype U112 or *fopC* mutant in the presence or absence of 1 ng/ml rIFN-γ and numbers of viable bacteria at 24 h determined by lysing the macrophages and dilution plating. Results are expressed as the averages of values for three wells. (B) C57BL/6 macrophages (10^6^/well) were infected (10 MOI) with U112 or *fopC* strain in the presence or absence of rIFN-γ (1 ng/ml) for 3 h. Protein-matched lysates from 3 h cultures with and without added rIFN-γ were resolved by SDS PAGE and analyzed by Western blotting with phosphotyrosine-STAT1 antibody or actin antibody. (C) Nitric oxide concentration was determined in the 24 h supernatants using Griess reagent. Differences between NO levels of U112 and *fopC* strains are significant with *p* values <0.001 as represented by * in the figure. (Representative of three experiments.)

Mononuclear phagocytes are an important component of innate defenses against *F. tularensis* [1] and a primary site for intracellular bacterial replication [23]. Thus, we compared replication of wildtype U112 and *fopC* KKF332 strains in C57BL/6 and IFN-γR^−/−^ macrophages, previously stimulated with rIFN-γ in order to better understand how IFN-γ signaling may control bacterial replication. Bone marrow derived primary macrophages (BMDM, 2×10^5^/well) prepared from C57BL/6 and IFN-γR^−/−^ mice were infected (MOI 10 or 100) with either U112 or KKF332 in the presence or absence of 1 ng/ml rIFN-γ and viable bacteria within the macrophages recovered at 3 and 24 h after inoculation. Since initial (3 h) bacterial uptake/replication was comparable under all conditions tested (data not shown), our studies were based on comparisons carried out at 24 hours. Both wildtype and *fopC* mutant bacteria replicated comparably in C57BL/6 and IFN-γR^−/−^ BMDM in the absence of rIFN-γ (Fig. 5A, 10 MOI). However, addition of rIFN-γ to C57BL/6 BMDM resulted in greater inhibition of intracellular *fopC* mutant replication (2.5–3.0 log_10_ reduction) than intracellular U112 replication (1.0–1.5 log_10_ reduction). Importantly, rIFN-γ mediated inhibition in BMDM was not observed in IFN-γR^−/−^ macrophages, demonstrating that activation of the IFN-γ signaling pathway is responsible for the observed inhibition of bacterial replication. Moreover, these results also indicate that the *fopC* mutant is more susceptible to IFN-γ-mediated killing within BMDM, when compared to the U112 parental strain. Addition of rIFN-γ to C57BL/6 BMDM also resulted in increased inhibition of *fopC* mutant replication compared to the wildtype U112 at 100 MOI (data not shown).

Recently, it has been reported that *F. novicida* downregulates the IFN-γ signaling pathway to evade host immunity [24]. Because we observed greater inhibition of replication of the *fopC* mutant than of the wildtype strain within rIFN-γ treated macrophages, we sought to determine the effect of the *fopC* mutant on the IFN-γ signaling pathway by examining phosphorylation of STAT1 (signal transducer and activator of transcription-1), a critical molecule in the IFN-γ pathway that binds and activates transcription of responding genes (such as *iNOS*), as well as at the levels of nitric oxide (NO) produced in BMDM infected with either the wildtype U112 or the *fopC* mutant. Minimally phosphorylated STAT1 (p-STAT1) was observed in mock infected BMDM or those infected with either wildtype or *fopC* mutant strain in the absence of rIFN-γ treatment (Fig. 5B); whereas, addition of rIFN-γ induced p-STAT1 in mock-infected BMDM. However, despite addition of rIFN-γ to BMDM infected with the wildtype U112 strain, phosphorylation of STAT1 was greatly suppressed (Fig. 5B) in agreement with previous studies from Roth and Parsa [24], [25]. In contrast, rIFN-γ treatment of BMDM infected with the *fopC* mutant resulted in p-STAT1 levels similar to that seen in rIFN-γ treated mock infected cells.

Given that STAT1 activation can result in induction of NO, we measured NO levels in culture supernatants of infected cells as previously described. We observed significantly increased NO production in rIFN-γ-treated BMDM infected with the *fopC* mutant compared to rIFN-γ-treated cells infected with wildtype U112 (Fig. 5C). Increased NO levels following rIFN-γ treatment in *fopC* mutant infected BMDM is all the more significant given that the level of NO induced in the absence of IFN-γ by *fopC* was significantly lower than in BMDM infected with U112 (Fig. 5C). These results suggest that the increased levels of NO production in rIFN-γ treated BMDM may contribute to the greater inhibition of replication observed for the *fopC* mutant as compared to the wildtype strain. Collectively, these results suggest a role for FopC in evading host IFN-γ-mediated responses.

### Characterization of the *fopC* mutant

Based upon predicted Signal Peptidase II cleavage site data, FopC is considered to be a lipoprotein [26]. Membrane lipoproteins can serve as structural proteins, enzymes, transporters, or immune evasion molecules [27]. The *fopC* mutant, KKF332, replicated similarly to U112 in both supplemented TSB and Chamberlain's medium within a 36 h growth period, and exhibited similar sensitivity to several classes of antibiotics (Table S1). However, biochemical characterization of KKF332 using substrate embedded API-ZYM strips revealed aberrant localization of acid phosphatase activity in the *fopC* mutant (Table S2). Acid phosphatase activity was observed associated with the whole cell fraction in the wildtype U112 strain; whereas, minimal acid phosphatase activity was found in the culture supernatant. In striking contrast, acid phosphatase activity of the *fopC* mutant was observed predominantly in the culture supernatant, and less associated with the whole cell fraction. This suggests that FopC contributes to the normal localization of acid phosphatases.

**Figure 6:**
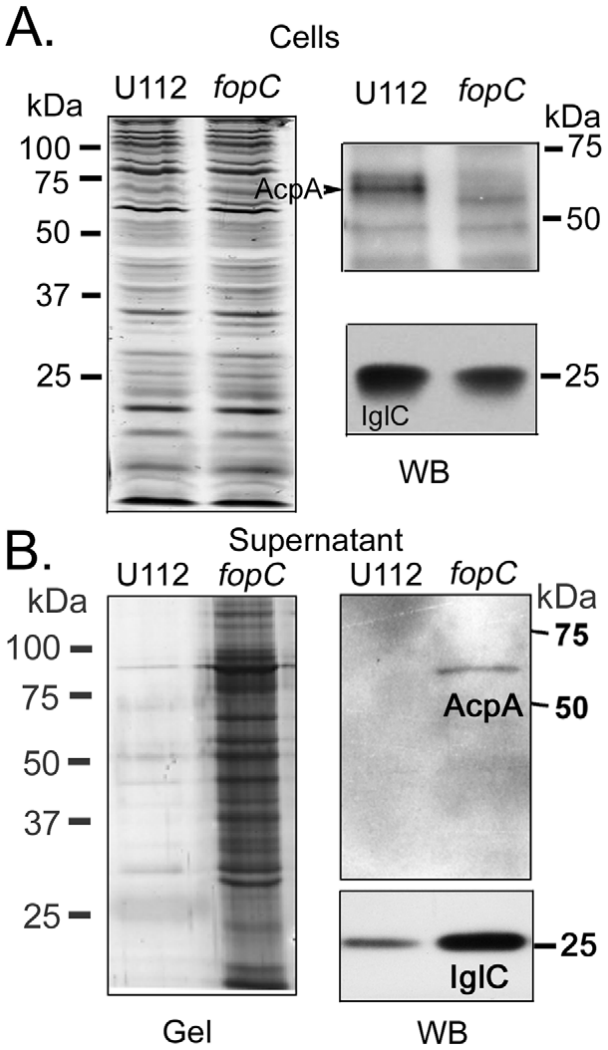
Biochemical characterization of *fopC* mutant phenotype. Total proteins from similarly prepared cell pellets (A) and concentrated (60 fold) spent (36 h) culture supernatant (B) of U112 and *fopC* mutant strain were separated by 10% SDS-PAGE and either stained by silver reagents (Gel) or transferred onto a PVDF membrane and probed with anti-AcpA antibody or anti-IglC antibody (WB).

**Figure 7:**
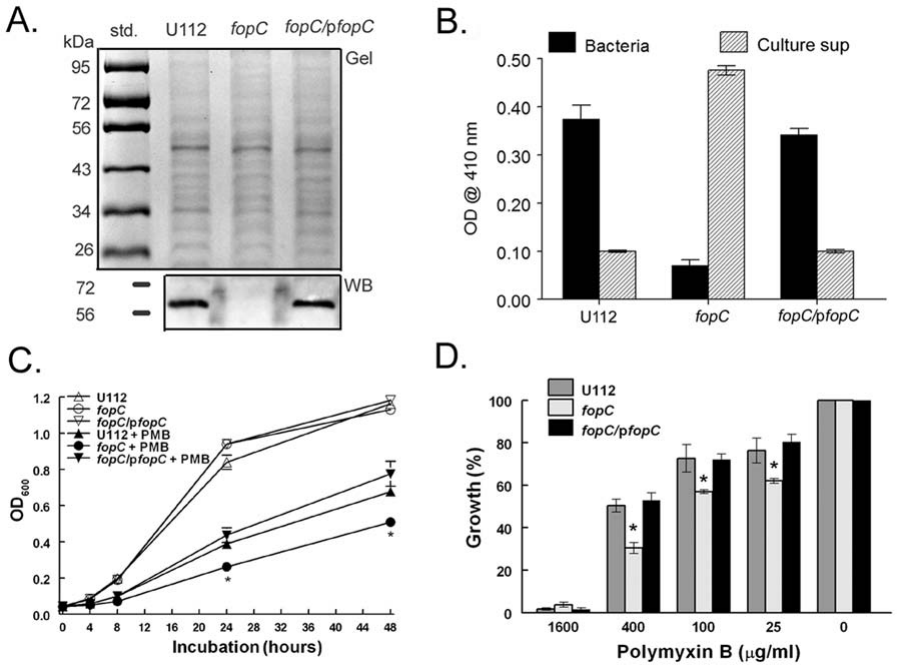
Acid phosphatase activity and resistance to polymyxin B treatment are restored in a *fopC-*complemented strain. (A) U112, *fopC* mutant and complemented *fopC*/p*fopC* cell lysates were separated by 10% SDS-PAGE and either stained with Coomassie blue (Gel) or transferred onto a PVDF membrane and probed with polyclonal FopC antisera (WB). (B) U112, *fopC* and *fopC*/p*fopC* were grown for 36 h in Chamberlain's medium. Bacteria and respective concentrated (60 fold) culture filtrates were analyzed for acid phosphatase activity using *p*-nitrophenylphosphate substrate. (C) Growth curves of U112, *fopC* mutant and complemented *fopC*/p*fopC* cells in Chamberlain's medium ± polymyxin B (400 µg/ml) over 48 h. *Difference in growth between *fopC* mutant and U112/complement strain was significant at a *p*<0.01 (D) Inhibition of U112, *fopC* mutant and complemented *fopC*/p*fopC* cell growth in Chamberlain's medium at 24 h by various concentration of polymyxin B. Bacterial growth in the presence of polymyxin B is presented as % to corresponding bacterial strain grown in the absence of polymyxin B. *Difference in growth between *fopC* mutant and U112/complement strain was significant at a *p*<0.01. (Representative of two experiments with triplicates for B–D.)

Currently, five acid phosphatase genes have been identified within the U112 genome and among these, AcpA is the major acid phosphatase associated with the outer membrane [28]. In order to determine if FopC is involved in proper localization of AcpA, Western immunoblot analyses utilizing AcpA antisera were carried out on U112 and KKF332 cell pellets and filtered culture supernatants. These analyses revealed that AcpA was more abundant in U112 pellets than in *fopC* mutant (Fig. 6A, WB). In contrast, AcpA was only found in the supernatant of the *fopC* mutant strain, but not in the supernatant of the wildtype U112 strain (Fig. 6B, WB). The presence of AcpA in the *fopC* mutant culture filtrate was confirmed further by proteomic analysis of the filtrate using Matrix Assisted Laser Desorption/Ionization-Time of Flight mass spectrometry (Table S3). Moreover, there was a greater abundance of different proteins in the supernatant of the *fopC* mutant strain than in the supernatant of the wildtype U112 strain (Fig. 6B, Gel); although growth conditions and CFUs used to generate the respective supernatants were similar. Some of the proteins (Table S3) in the *fopC* culture supernatant were identified by proteomic analysis with molecular weights ranging from 9 kDa to 100 kDa. These proteins consist of putative cytoslic proteins (e.g. DNA-binding protein HU-beta, isocitrate dehydrogenase), periplasmic proteins (e.g. beta-lactamase class A), membrane proteins (OmpA family protein, AcpA), and previously identified secreted proteins (ChiA, ChiB, CpbA, PepO) [29] suggesting proteins were released into culture through the “leaky” membrane of the *fopC* mutant. Western blot analysis of another identified protein, IglC, also revealed similar protein localization pattern to AcpA (Fig. 6A and 6B). These results suggest that FopC protein may play a role in maintaining outer membrane stability (integrity) since in the absence of FopC, acid phosphatases (especially AcpA) and a variety of other proteins normally cell associated could now be found in the supernatant. To further confirm that the membrane of the *fopC* mutant is less stable than the parental U112 strain, we compared the susceptibility of both bacterial strains to polymyxin B (PMB) treatment. Polymyxin B is a cationic cyclic peptide with a fatty acid tail, which interacts with Gram-negative bacterial membranes and affects membrane stability [30], [31], [32]. As shown in Fig. 7C, the *fopC* mutant replicated in a similar manner to U112 in the absence of PMB, but demonstrated significantly (*p*<0.01) slower growth in the presence of 400 µg/ml PMB at 24 and 48 h after bacterial inoculation. Moreover, the sensitivity of *F. novicida* to PMB was dose-dependent, and the *fopC* mutant exhibited greater sensitivity to PMB than U112 even at a low concentration (25 µg/ml [*p*<0.01], Fig. 7D) suggesting decreased *F. novicida* membrane stability in the absence of the FopC protein.

### Complementation of KKF332 with the *fopC* gene restores virulence

**Figure 8:**
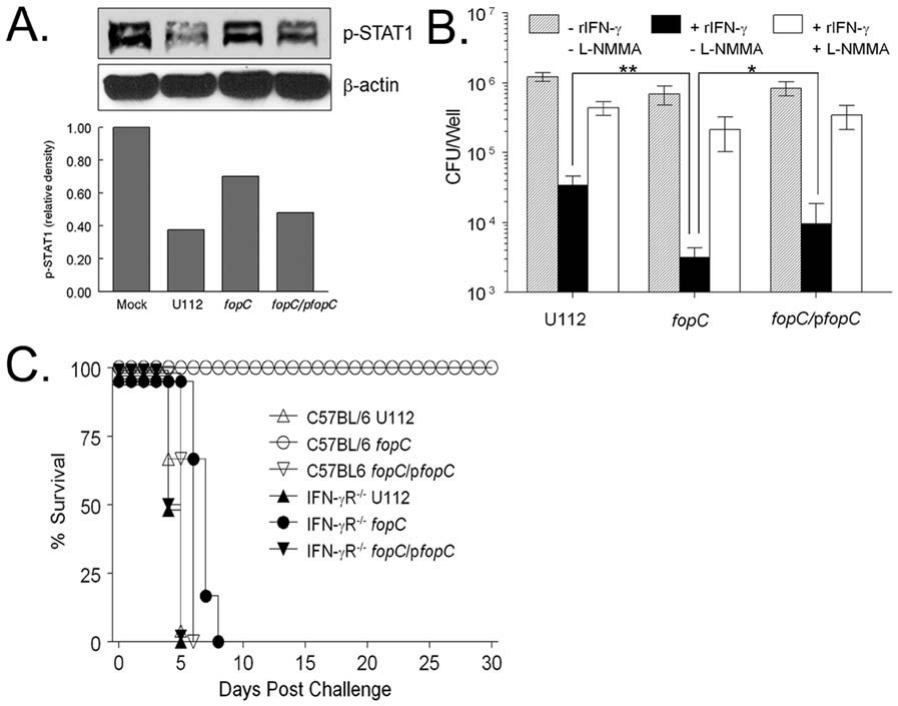
Virulence is restored in a *fopC-*complemented strain. (A) C57BL/6 macrophages (10^6^/well) were infected (10 MOI) with U112, *fopC*, or complemented *fopC*/p*fopC* strain in the presence or absence of rIFN-γ (1 ng/ml) for 3 h. Protein-matched lysates from 3 h cultures were resolved by SDS PAGE and analyzed by Western blotting with phosphotyrosine-STAT1 and actin antibodies. The relative p-STAT1 levels of the three strains were first normalized with respective β-actin level and compared to mock treated p-STAT1. (B) Bone marrow derived primary macrophages (2×10^5^/well) from C57BL/6 or IFN-γR^−/−^ mice were infected (10 MOI) with wildtype U112, *fopC* mutant or *fopC*/p*fopC* in the presence or absence of 1 ng/ml rIFN-γ and 0.25 mM NOS inhibitor L-NMMA, and numbers of viable bacteria at 24 h determined by macrophage lysing and dilution plating. Results are expressed as the averages of values for three wells. Differences in bacterial replication within IFN-γ activated macrophages between *fopC* mutant and U112** or *fopC*/pfopC* were significant at a *p*<0.01** and *p*<0.05*, respectively. (C) C57BL/6 and IFN-γR^−/−^ mice (n = 6/group) were challenged i.n. with 10^3^ CFU U112, mutant *fopC* (KKF332) strain, or KKF332 strain complemented with p*fopC* (*fopC/*p*fopC*). Difference in survival in challenged BALB/c mice between *fopC* mutant and complement strain was significant at a *p*<0.001. All animals were monitored daily for survival for 30 days. (Representative of two-three experiments.)

In order to confirm that phenotypes associated with the KKF332 strain are due only to the loss of FopC, we transformed this strain with a plasmid (p*fopC*) that expresses *fopC* from its native promoter. Expression of FopC in the complemented strain (*fopC*/p*fopC*) was confirmed by Western blot analysis using anti-FopC antibody (Fig. 7A). Given the marked difference in acid phosphatase localization between the wildtype U112 and *fopC* mutant strains, the cell pellet and culture supernatant material of p*fopC* complemented strain was analyzed for localization of acid phosphatase. As previously shown, wildtype U112 acid phosphatase activity is predominantly cell associated with little activity in the culture supernatant; whereas, acid phosphatase activity of the *fopC* mutant strain is observed to be predominantly in the supernatant, with little cell associated activity (Fig. 7B). When *fopC* KKF332 was complemented with pf*opC*, localization of acid phosphatase activity resembled that of the wildtype strain, i.e., high cell-associated levels of activity, in contrast to low activity levels observed in the culture supernatant (Fig. 7B). Furthermore, resistance to PMB damage was restored in the *fopC* complemented (*fopC*/p*fopC*) strain similar to the level of wildtype U112 (Fig. 7C and D). Complementation of *fopC* mutant with p*fopC* also restored inhibition of p-STAT1 to a level similar to wildtype U112 in IFN-γ stimulated BMDM (Fig. 8A), and restored intramacrophage replication in IFN-γ treated BMDM to a level similar to that observed for the wildtype strain (Fig. 8B). The inhibition of *fopC* mutant replication in IFN-γ treated BMDM might largely be due to production of nitric oxide, an effector of IFN-γ signaling since addition of 0.25 mM N^G^-monomethyl-L-arginine (L-NMMA), an NOS inhibitor, led to an increase of *fopC* replication to a level similar to that in the BMDM unstimulated with rIFN-γ (Fig. 8B). Finally, p*fopC* complementation restored virulence. C57BL/6 and IFN-γR^−/−^ mice (6/group) were challenged i.n. with 10^3^ CFU U112, the *fopC* mutant (KKF332) strain, or complemented KKF332 carrying p*fopC* (*fopC*/p*fopC*); and monitored for 30 days. As shown in Fig. 8C, all C57BL/6 mice succumbed to infection with wildtype U112 within five days; whereas, all mice survived infection with the *fopC* strain in agreement with the results presented above. Importantly, complementation of the *fopC* mutant with p*fopC* fully restored virulence (*p*<0.001 compared to *fopC* mutant challenged mice), with all mice succumbing to infection by day 6. Additionally, IFN-γR^−/−^ mice infected with U112, the *fopC* mutant (KKF332) strain, or KKF332 complemented with p*fopC* succumbed to infection within eight days of infection as expected and consistent with earlier results. These results demonstrate that the attenuated virulence phenotype of KKF332 is specifically dependent upon loss of FopC. Thus, complementation of the KKF332 mutant with p*fopC* restored acid phosphatase activity localization, inhibition of IFN-γ-mediated responses, as well as levels of virulence observed for the wildtype organism in the murine pulmonary tularemia model.

## Discussion

*Francisella*, as with other obligate intracellular and facultative intracellular bacteria, has evolved various strategies to evade early host immune defenses including inhibition of phagolysosomal fusion [33], [34] and early acidification [4], [35], [36]. Once the organism escapes the phagosome, it replicates in the host cytosol and induces apoptosis and lysis of infected cells. Additionally, the infected host cells and extracellular bacteria lead to dissemination of *Francisella* from the lung during pulmonary infection. In this report, we describe the role of a *F. novicida* outer membrane protein, FopC, in evasion of IFN-γ signaling and virulence of this organism in an experimental mouse model.

Evasion of IFN-γ-mediated host immune defenses has been characterized for a number of viruses [37], [38] as well as for several intracellular bacteria [39], [40], [41]. For example, *Salmonella typhimurium* evades killing in activated macrophages by inhibition of IFN-γ-induced nitric oxide production. This evasion is dependent on the membrane associated protein NirC, while *Chlamydia trachomatis* employs a different mechanism to evade IFN-γ-mediated host defense to achieve persistent infection [42]. IFN-γ-mediated indoleamine 2,3-dioxygenase upregulation and tryptophan starvation affect the developmental cycle of *Chlamydia trachomatis* [42]. In the presence of exogenous indole, expression of functional chlamydial tryptophan synthase has been shown to effectively overcome IFN-γ-mediated killing [43]. In contrast, *F. tularensis* infections are more acute and rapidly fatal. Tridandapani and colleagues [24] have shown that *F. novicida* infection of murine macrophages results in suppression of IFN-γ-induced STAT1 expression and phosphorylation. Our results now demonstrate a plausible mechanism by which bacterial components are involved in the *F. novicida*-mediated inhibition of IFN-γ-mediated signaling. IFN-γ-treated primary macrophages exposed to the *fopC* mutant exhibited greater levels of p-STAT1 as early as 3 h compared to the wildtype strain. Moreover, *fopC* mutant-infected macrophages also produced greater NO in the supernatant, and exhibited greater inhibition of intracellular bacterial replication. This inhibition of *fopC* replication was abrogated by the presence of a NOS inhibitor suggesting that the IFN-γ signaling effector, nitric oxide, was important for control of *fopC* mutant infection.

During phenotypic characterization of the *ΔfopC* with API-ZYM, we noticed that acid phosphatase activity, which is normally cell-associated in the wildtype strain, was largely found in culture supernatants of the *fopC* mutant. This raises the question whether the aberrant acid phosphatase phenotype of the *fopC* mutant plays a role in modulation of IFN-γ signaling. It is quite plausible based on (1) precedential evidence supporting the involvement of phosphatase(s) in inhibition of IFN-γ signaling; for example, Vaccina virus inhibits IFN-γ signaling via virus-associated phosphatase activity (VH1) [37]; (2) a recent publication showing *F. novicida* AcpA was capable of dephsophorylating other host defense proteins [44]; and (3) our observation of macrophages infected with a strain lacking four acid phosphatases (including AcpA) showing no impairment of p-STAT1 formation, similar to the *fopC* mutant (data not shown).

One interesting phenotype of the *fopC* mutant strain is that many proteins are present in the culture supernatant of the *ΔfopC* strain that are either absent or present at much lower levels in supernatants from the wildtype strain. This suggests that FopC functions to stabilize the outer membrane and that its absence leads to outer membrane destabilization and release of multiple membrane-associated proteins such as AcpA. Additional evidence in the published literature appears to support membrane-stabilization by FopC. Previous studies of Lindgren *et al*. [45] demonstrated that a FTT0918 (*fopC*) mutant of *F. tularensis* subsp. *tularensis* exhibits an inability to acquire iron bound to siderophores. We suggest that destabilization of the outer membrane in the absence of FTT0918 (FopC) might lead to loss of the siderophore receptor, thus resulting in the observed iron deficient phenotype. Experiments are currently being carried out to test this hypothesis.

In summary, findings presented here indicate that the FopC protein of *F. novicida* is a virulence determinant and functions to maintain membrane integrity and the proper localization of factors such as AcpA which may be one of several *F. novicida* bacterial components involved in the dephosphorylation of STAT1 to inhibit IFN-γ-mediated signaling. Together, our findings support the importance of FopC in facilitating avoidance of immunosurveillance and promoting intracellular bacterial growth.

## Materials and Methods

### Bacteria

*Francisella novicida* strain U112 was provided by Dr. Francis Nano (University of Victoria, Canada). The *F. novicida* FTN_0444 (FopC) insertion mutant of U112 was generated using a Targetron transformation system as described previously [18]. Briefly, U112 was transformed using plasmid pKEK1220 (372|373s) via electroporation (600Ω, 25 µF, 2.5 kV; BIO-RAD Gene Pulser II). The pKEK1220 plasmid was constructed by retargeting the *Ll.LtrB* intron in pKEK1140 using intron binding site primer IBS (5′-AAAACTCGAGATAATTATCCTTAAAAAACCCTCGTGTGCGCCCAGATAGGGT G), and two exon binding site primers EBS1 (5′-CAGATTGTACAAATGTGGTGATA ACAGATAAGTCCCTCGTACTAACTTACCTTTCTTTGT) and EBS2 (5′-TGAACGCAAGTTTCTAATTTCGGTTTTTTTCCGATAGAGGAAAGTGTCT) as described previously [18]. The *fopC* mutant, KKF332 (FTN_0444:: *Ll.LtrB*), was obtained by PCR screening using gene-specific and intron-specific primers and further verified by DNA sequencing and Southern analysis. Complemented strain *ΔfopC*/p*fopC* was generated *via* electroporation (as described above) using the complementation plasmid (p*fopC*) containing *fopC* coding sequences plus its putative promoter (∼200 bp upstream) PCR amplified from U112 DNA using gene specific primers (5′-CGGAATTCCATGGACACCGACGTATGCAC and 5′-AAGAAAAAAGCGGCCGC TTATACGTATACCGACATATCCAG).

### Mice

Female mice were used in all experiments. Four to six week old BALB/c and C57BL/6 mice were purchased from the National Cancer Institute (Bethesda, MD). BALB/c IFN-γ^−/−^ [46] and C57BL/6 MHC I (β2m)^−/−^ mice [47], MHC II (H2)^−/−^ mice [48], µMT (B-cell deficient) mice [49] and IFN-γR^−/−^ [50] mice were purchased from the Jackson Laboratory (Bar Harbor, ME, USA). Mice were housed at the University of Texas at San Antonio animal facility and all experimental procedures were approved by the UTSA Institutional Animal Care and Use Committee (IACUC: MU031-11/11A7).

### Intranasal challenge and bacterial dissemination

Mice were first anesthetized with 3% isoflurane, using a rodent anesthesia system (Harvard Apparatus, Holliston, MA), and then inoculated intranasally (i.n.) with respective doses of *F. novicida* strains in 25 µl of phosphate-buffered saline (PBS). The actual CFU administered in each experiment was determined by dilution plating of inocula on supplemented TSA. For survival experiments, animals were monitored daily for morbidity and mortality. For bacterial dissemination assays, mice were challenged i.n. with 10^3^ CFU of U112 (lethal dose) or KKF332 (sublethal dose). At 1, 2, and 3 days postchallenge with U112 infected mice and at 1, 2, 3, 7, and 14 days with KKF332 infected mice (3 mice at each time point), lungs, livers, and spleens were collected into 3 ml of Dulbecco's modified Eagle's medium containing 4.5 g/L glucose, L-glutamine, sodium pyruvate (DMEM; Mediatech, Fairfax, VA) and supplemented with 10% (v/v) fetal bovine serum (D10; HyClone, Logan, UT), homogenized, adjusted to a final volume of 5 ml and dilution plated on supplemented TSA for bacterial enumeration. Survival data indicate no significant differences between the two strains (BALB/c and C57BL/6) of mice used in this study with regard to U112 or *fopC* mutant susceptibility. None of the U112-infected mice survived past day 7.

### Antibodies

For generation of polyclonal antisera against FopC, 10 µg recombinant FopC protein was emulsified in an equal volume of complete Freund's adjuvant and injected intraperitoneally into 4 to 6-week-old, female BALB/c mice. After 2 weeks, mice were boosted with the same amount of protein with incomplete Freund's adjuvant. Anti-FopC sera were prepared 2 weeks post boost. Antibodies specific for phosphotyrosine-STAT1 were purchased from Cell Signaling Technology (Beverly, MA). β-actin antibody was purchased from Sigma-Aldrich (St. Louis, MO). AcpA antiserum was a gift from Dr. Thomas Reilly (University of Missouri).

### Infection of macrophages

Primary macrophages were prepared from murine bone marrow cells as previously described [51]. These bone marrow derived macrophages (BMDM) were washed with DMEM and resuspended to a density of 10^6^ cells/ml in D10. For intramacrophage growth assays, the suspension was aliquoted in 200 µl volumes per well in 96-well tissue culture plates and incubated for 2 h with U112 or KKF332 bacteria at a multiplicity of infection (MOI) of 10 or 100 bacteria/macrophage followed by 1 h gentamicin (GIBCO BRL, Grand Islands, N.Y.) treatment (20 µg/ml) to kill extracellular organisms. Wells were replenished with D10 and cells were grown at 37°C in a 5% CO_2_ incubator for an additional 21 h. Measurements of intramacrophage bacteria at 24 h post inoculation were performed as previously described [52]. In IFN-γ signaling assay experiments, BMDM were seeded into 6-well plates at a concentration of 10^6^ cells/well in 1 ml D10 overnight, and treated with 1 ng/ml rIFN-γ (eBioscience) for 2 h followed by inoculation with 10 MOI wildtype or mutant bacteria. A dose titration experiment was initially carried out to determine the optimal IFN-γ concentration for intramacrophage replication and 1 ng/ml concentration was used in subsequent IFN-γ signaling studies because of the maximal (but not saturated) effect on control of *ΔfopC* replication. BMDM were lysed after 3 h incubation by adding 100 µl SDS sample buffer (62.5 mM Tris-HCl, pH 6.8, 2% w/v SDS, 10% glycerol, 50 mM DTT, 0.01% w/v bromophenol blue) per well, sonicated for 15–20 sec, and centrifuged (12,000×g, 10 min). Culture filtrates (30 µl) of these cells were analyzed for p-STAT1 by Western blotting using monoclonal antibody per manufacturer's recommendations (Cell Signaling Technology).

### API-ZYM and acid phosphatase assays

U112 or mutant strains were grown with an inoculum of 100 µl (OD_600_ = 0.5) in 30 ml Chamberlain's medium [53] for 36 hrs. Bacteria were collected by centrifugation and adjusted to an OD_600_ of 0.3. The culture supernatants (following centrifugation) were filtered through 0.22 µm filters, and concentrated to 0.5 ml using a 10,000 MW cut-off Centripreps (Millipore, Billerica, MA). Bacteria (65 µl) or the concentrated culture filtrates were added to API-ZYM strips (bioMeriux, Inc, Durham, NC), incubated for 4 h at 37°C and analyzed for enzymatic profiles according to manufacturer's instructions. Quantitative acid phosphatase activity was determined by release of *p*-nitrophenol (pNP) from *p*-nitrophenylphosphate substrate (pNPP; Sigma Chemical, St. Louis, Mo.) [54]. Briefly, 20 µl concentrated culture filtrate or OD_600_ = 1 of bacteria were added to 100 µl 50 mM citric acid buffer (pH 5) containing 7.6 mM pNPP in a 96-well plate, incubated at 37°C for 5 h, and the production of pNP measured at 410 nm using a microplate reader (µQuant; Biotek Instruments, Winooski, VT).

### Determination of nitrite concentration

The concentration of nitrite in cell supernatants was estimated using the Griess reagent [55]. Briefly, 100 µl supernatant was mixed with 50 µl Griess reagent and incubated at room temperature in the dark for 30 min. Absorbance was measured at 520 nm and nitrite concentrations determined by extrapolation using a NaNO_2_ standard curve (1–100 µM).

### Inhibition of bacterial growth by polymyxin B

Log phase *F. novicida* bacterial strains were used to inoculate 200 µl Chamberlain's medium (to an OD_600_ = 0.05) per well containing various concentration of polymyxin B sulfate salt (0–1.6 mg/ml, Sigma) in 96 well polystyrene microplates. Bacteria were allowed to growth for up to 48 h in a 37°C incubator and the turbidity of bacterial cultures was measured at 600 nm at 4, 8, 24 and 48 h after inoculation using a microplate reader.

### Statistical analysis

SigmaStat (Systat Software Inc., San Jose, CA) was used to perform all tests of significance. Statistical analyses for survival experiments were performed using the Kaplan-Meier test. The Student's *t* test was used to determine differences in *in vitro* and *in vivo* bacterial replication. Data are all presented as mean values ± the respective standard deviation.

## Supporting Information

Table S1 Inhibition of *F. novicida* growth by various classes of antibiotics. *F. novicida* wildtype U112 and *fopC* mutant strain were grown to 0.6 OD_600_, diluted to a Mcfarland 1 standard and 100 µl of each culture was confluently spread onto Mueller-Hinton agar plates, and the antibiotic discs placed on the plates. The plates were incubated at 37°C overnight and the zones of inhibition in mm measured around each disc. Data presented above is the average of readings from three discs of each antimicrobial.
(DOCX)

Table S2 Enzymatic characterization of wildtype U112 and *fopC* mutant using the API-ZYM kit. * Intensity of the color reactions was graded from 0 to 5 according to an API-ZYM color reaction chart.
(DOCX)

Table S3 Proteins identified in the culture supernatant of the *fopC* mutant. The *fopC* mutant was grown in Chamberlain's medium for 36 h. Culture filtrate was concentrated using 10,000 MW cut-off Centripreps, digested with trypsin, and subjected to proteomic analysis using Matrix Assisted Laser Desorption/Ionization-Time of Flight mass spectrometry. Each of the identified proteins in this list contains at least 5 matched peptide spectra.
(DOCX)
