# Supplementary material for: Evasion of IFN-γ Signaling by Francisella novicida Is Dependent upon Francisella Outer Membrane Protein C
Source: PLoS One. 2011 Mar 31;6(3):e18201. doi: 10.1371/journal.pone.0018201 (PMC3069069; doi:10.1371/journal.pone.0018201)
Supplement: Table S1 — Inhibition of F. novicida growth by various classes of antibiotics. F. novicida wildtype U112 and fopC mutant strain were grown to 0.6 OD600, diluted to a Mcfarland 1 standard and 100 µl of each culture was confluently spread onto Mueller-Hinton agar plates, and the antibiotic discs placed on the plates. The plates were incubated at 37°C overnight and the zones of inhibition in mm measured around each disc. Data presented above is the average of readings from three discs of each antimicrobial. (DOCX) [file pone.0018201.s001.docx]

| Antibiotic | Function (inhibitor) | Concentration per disc | Diameter of Zone of Inhibition in mm | |
| --- | --- | --- | --- | --- |
|  |  |  | U112 | *fopC* |
| Bacitrcin | Cell wall biosynthesis | 2 IU | 0 | 0 |
| Methicillin | Cell wall biosynthesis | 5 mcg | 0 | 0 |
| Penicillin | Cell wall biosynthesis | 10 IU | 0 | 0 |
| Novobiocin | DNA gyrase | 5 µg | 25.3 ± 0.6 | 25.3 ± 0.6 |
| Sulfathiazole | Folic acid biosynthesis | 0.25 mg | 0 | 0 |
| Trimethoprim | Folic acid biosynthesis | 5 µg | 9.7 ± 0.6 | 10 |
| Chloromphenicol | Protein synthesis | 30 µg | 27.7 ± 0.6 | 27.3 ± 1.5 |
| Gentamycin | Protein synthesis | 5 µg | 20.7 ± 0.6 | 19.3 ± 0.6 |
| Streptomycin | Protein synthesis | 10 µg | 0 | 0 |
| Tetracycline | Protein synthesis | 30 µg | 17 ± 1 | 18 |
