# Supplementary material for: Evasion of IFN-γ Signaling by Francisella novicida Is Dependent upon Francisella Outer Membrane Protein C
Source: PLoS One. 2011 Mar 31;6(3):e18201. doi: 10.1371/journal.pone.0018201 (PMC3069069; doi:10.1371/journal.pone.0018201)
Supplement: Table S2 — Enzymatic characterization of wildtype U112 and fopC mutant using the API-ZYM kit. * Intensity of the color reactions was graded from 0 to 5 according to an API-ZYM color reaction chart. (DOCX) [file pone.0018201.s002.docx]

| Enzyme | Control | Alkaline Phosphatase | Esterase (C 4) | Esterase Lipase (C 8) | Lipase | Leucine arylamidase | Valine arylamidase | Cystine aramadylase | Trypsin | α- Chymotrypsin | **Acid Phosphatase** | Napthol-AS-BI-Phosphohydrlase | α - galactosidase | β- galactosidase | β- glucuronidase | α - glucosidase | β- glucosidase | N- acetyl – b- glucosaminidase | α - monosidase | β- monosidase |
| --- | --- | --- | --- | --- | --- | --- | --- | --- | --- | --- | --- | --- | --- | --- | --- | --- | --- | --- | --- | --- |
| U112 Cells | 0 | 2 | 2 | 4 | 0 | 3 | 0 | 1 | 1 | 1 | **5** | 2 | 0 | 0 | 0 | 0 | 1 | 1 | 1 | 1 |
| *fopC* Cells | 0 | 0 | 2 | 4 | 0 | 3 | 0 | 1 | 1 | 1 | **1** | 4 | 1 | 1 | 0 | 1 | 1 | 1 | 1 | 1 |
| U112 Supernatant | 0 | 0 | 2 | 0 | 1 | 0 | 0 | 0 | 0 | 0 | **1** | 1 | 2 | 2 | 2 | 2 | 2 | 2 | 2 | 2 |
| *fopC* Supernatant | 0 | 2 | 2 | 2 | 1 | 0 | 0 | 0 | 0 | 0 | **5** | 5 | 2 | 2 | 2 | 2 | 2 | 2 | 2 | 2 |
