# Supplementary material for: Evasion of IFN-γ Signaling by Francisella novicida Is Dependent upon Francisella Outer Membrane Protein C
Source: PLoS One. 2011 Mar 31;6(3):e18201. doi: 10.1371/journal.pone.0018201 (PMC3069069; doi:10.1371/journal.pone.0018201)
Supplement: Table S3 — Proteins identified in the culture supernatant of the fopC mutant. The fopC mutant was grown in Chamberlain's medium for 36 h. Culture filtrate was concentrated using 10,000 MW cut-off Centripreps, digested with trypsin, and subjected to proteomic analysis using Matrix Assisted Laser Desorption/Ionization-Time of Flight mass spectrometry. Each of the identified proteins in this list contains at least 5 matched peptide spectra. (DOCX) [file pone.0018201.s003.docx]

| FTN no. | Proteins | Molecular Weight |
| --- | --- | --- |
| FTN_0022 | histidine acid phosphatase | 39 kDa |
| FTN_0090 | acid phosphatase, AcpA | 58 kDa |
| FTN_0191 | hypothetical protein | 28 kDa |
| FTN_0275 | hypothetical protein | 40 kDa |
| FTN_0627 | chitinase, glycosyl hydrolase family 18 ChiA | 96 kDa |
| FTN_0633 | peroxidase/catalase | 82 kDa |
| FTN_0637 | glycerophosphoryl diester phosphodiesterase | 39 kDa |
| FTN_0756 | OmpA family protein | 41 kDa |
| FTN_0855 | hypothetical protein | 26 kDa |
| FTN_0869 | hypothetical protein | 73 kDa |
| FTN_0907 | D-alanyl-D-alanine carboxypeptidase | 48 kDa |
| FTN_0963 | NAD-dependent aldehyde dehydrogenase | 54 kDa |
| FTN_0980 | malate dehydrogenase | 34 kDa |
| FTN_1054 | DNA-binding protein HU-beta | 9 kDa |
| FTN_1072 | beta-lactamase class A | 32 kDa |
| FTN_1186 | M13 family metallopeptidase, PepO | 79 kDa |
| FTN_1260 | hypothetical membrane protein | 52 kDa |
| FTN_1284 | chaperone, heat shock protein, HSP 70 family | 69 kDa |
| FTN_1322 | intracellular growth locus C protein, IglC | 22 kDa |
| FTN_1332 | glyceraldehyde-3-phosphate dehydrogenase | 35 kDa |
| FTN_1434 | isocitrate dehydrogenase | 82 kDa |
| FTN_1448 | hypothetical protein | 52 kDa |
| FTN_1485 | chitin-binding protein, CpbA | 63 kDa |
| FTN_1494 | pyruvate dehydrogenase subunit E1 | 100 kDa |
| FTN_1532 | glutamate dehydrogenase | 49 kDa |
| FTN_1538 | chaperonin GroEL | 57 kDa |
| FTN_1744 | chitinase, ChiB | 79 kDa |
